# Immunometric and functional measurement of endogenous vasoinhibin in human sera

**DOI:** 10.3389/fendo.2024.1345996

**Published:** 2024-04-29

**Authors:** Magdalena Zamora, David Harris, Nils Davies, Johannes Ebnet, Peter Radermacher, Cosima Brucker, Christiane Waller, Juan Pablo Robles, Thomas Bertsch, Carmen Clapp, Jakob Triebel

**Affiliations:** ^1^ Institute for Clinical Chemistry, Laboratory Medicine and Transfusion Medicine, Nuremberg General Hospital & Paracelsus Medical University, Nuremberg, Germany; ^2^ Instituto de Neurobiología, Universidad Nacional Autónoma de México (UNAM), Querétaro, Mexico; ^3^ Institute of Anesthesiological Pathophysiology and Process Engineering, University of Ulm, Ulm, Germany; ^4^ Department of Gynecology and Obstetrics, Nuremberg General Hospital & Paracelsus Medical University, Nuremberg, Germany; ^5^ Department of Psychosomatic Medicine and Psychotherapy, Nuremberg General Hospital & Paracelsus Medical University, Nuremberg, Germany

**Keywords:** prolactin, vasoinhibin, 16 kDa prolactin, 16K prolactin, ELISA, preeclampsia

## Abstract

**Introduction:**

Circulating levels of the antiangiogenic protein vasoinhibin, a fragment of prolactin, are of interest in vasoproliferative retinopathies, preeclampsia, and peripartum cardiomyopathy; however, it is difficult to determine the circulating levels of vasoinhibin due to the lack of quantitative assays.

**Methods:**

This study used human serum samples to assess the concentration and bioactivity of vasoinhibin using a novel enzyme-linked immunosorbent assay (ELISA) for human vasoinhibin, which employs an anti-vasoinhibin monoclonal antibody, a human umbilical vein endothelial cell (HUVEC) proliferation assay, and a chick chorioallantoic membrane (CAM) angiogenesis assay.

**Results:**

Serum samples from 17 pregnant women without (one group) and with preeclampsia and pregnancy induced hypertension (another group) demonstrated endogenous vasoinhibin concentrations in the range of 5–340 ng/ml. Immunoactive vasoinhibin levels were significantly higher in preeclampsia serum compared to healthy pregnancy serum (mean 63.09 ± 22.15 SD vs. 19.67 ± 13.34 ng/ml, p = 0.0003), as was the bioactive vasoinhibin level as determined by the HUVEC proliferation assay (56.12 ± 19.83 vs. 13.38 ± 4.88 ng/ml, p < 0.0001). There was a correlation between the concentration of vasoinhibin measured by ELISA and the HUVEC proliferation assay (Pearson r = 0.95, p < 0.0001). Healthy serum demonstrated a proangiogenic effect in the CAM assay (p < 0.05, compared to control), while serum from preeclamptic patients demonstrated an antiangiogenic effect (p < 0.05 vs. control), as did recombinant human vasoinhibin and a synthetic circular retro-inverse vasoinhibin analogue (CRIVi45-51). The antiangiogenic effects in the CAM assay and the inhibition of HUVEC proliferation were abolished by addition of the ELISA anti-vasoinhibin monoclonal antibody, but not by mouse IgG.

**Discussion:**

These results demonstrate the first quantitation of endogenous vasoinhibin in human sera and the elevation of it levels and antiangiogenic activity in sera from women with preeclampsia. The development and implementation of a quantitative assay for vasoinhibin overcomes a long-standing barrier and suggests the thorough clinical verification of vasoinhibin as a relevant biomarker.

## 1 Introduction

The antiangiogenic protein hormone vasoinhibin, which is generated by the proteolytic cleavage of prolactin (PRL), is of interest in the context of various diseases, including vasoproliferative retinopathies and diabetic macular oedema (1, 2), peripartum cardiomyopathy (3–5), preeclampsia (6–8), inflammatory arthritis (9, 10), and cancer (11, 12). The interest is mainly due to the vascular actions of vasoinhibin, which include the inhibition of endothelial cell proliferation, vasodilation, and vasopermeability, all relevant in the above mentioned diseases (13). Cathepsin D is the primary protease responsible for the generation of vasoinhibin in adenohypophyseal PRL secretory granules and cathepsin D-null mice (homozygous mice in which the cathepsin D gene was disrupted) are devoid of hypophyseal vasoinhibin (14). It is also generated in peripheral tissues (15). A high affinity cleavage site in PRL for the generation of vasoinhibin, which emerged in Simians and is conserved in higher primates, appears as a gain-of-function event under positive selection (16). Vasoinhibin is present in both, males and females. A high-affinity binding site on endothelial cells was reported (17), and microRNA-146a (18), plasminogen-activator inhibitor-1 (PAI-1) (19), and integrin alpha5 beta1 (20) have been described as messengers and interaction partners, respectively.

Circulating vasoinhibin, previously also designated as 16 kDa PRL or 16K PRL (21), is usually detected by immunoprecipitation followed by sodium dodecyl-sulfate polyacrylamide gel electrophoresis (SDS-PAGE) and Western blotting; however, there are disadvantages to this method, which include limited sensitivity, its semi-quantitative value, and dependence on polyclonal antibodies with limited specificity. Other techniques that have been occasionally reported include a lab-on-a-chip based method employing laser induced-fluorescence and a mass spectrometric approach; however, neither of these methods have produced quantitative analyte concentrations and are not suitable for routine applications (22–24). Therefore, the development of a quantitative assay for the quantitation of vasoinhibin in human blood samples, such as an immunometric assay, is required.

Preeclampsia is a significant cause of maternal and neonatal morbidity and mortality. The syndrome manifests with hypertension and proteinuria, and develops into a poorly perfused fetoplacental unit with abnormal blood vessels and fetal growth restriction (25). Preeclampsia is defined as elevated blood pressure during pregnancy as ≥ 140 mmHg systolic or 90 mmHg diastolic and proteinuria ≥ 300 mg per 24-hour urine collection (more detailed diagnostic criteria can be reviewed in ref (25)). The presence of the placenta, as opposed to the fetus, is essential for the development of preeclampsia; the shedding of antiangiogenic factors from the placenta into the maternal circulation contributes to maternal endothelial dysfunction (26). Clinical studies have reported increased levels of vasoinhibin in the circulation, urine, and amniotic fluid of patients with preeclampsia, and suggested that it may contribute to endothelial cell dysfunction in preeclampsia (6–8, 27). In this context, the chick chorioallantoic membrane (CAM) angiogenesis assay is a relevant model. Not only has it been traditionally used to investigate the antiangiogenic effects of vasoinhibin (28, 29), by being considered the avian homologue of the mammalian placenta, the CAM assay is also a valuable model for the study of human reproduction and its complications. This particularly concerns the study of natural blood vessel growth and function, as the CAM’s intermediate mesodermal layer is highly vascularized, rich in stromal components, and accessible to observation, manipulation, and analysis (30).

In the present study, we report the immunometric measurement of endogenous vasoinhibin in the serum of pregnant women without and with preeclampsia using a novel enzyme-linked immunosorbent assay (ELISA) with an anti-vasoinhibin monoclonal antibody (vi-mab), which shows no cross-reactivity with PRL. The development of such antibodies was recently reported (31). Furthermore, the specificity of the ELISA is supported by its strong correlation with the bioactive levels of vasoinhibin determined by the inhibition of human umbilical vein endothelial cell (HUVEC) proliferation, which is prevented by vi-mab. Finally, by using the CAM angiogenesis assay, we further confirmed the high levels of vasoinhibin in serum from preeclamptic women, supporting its antiangiogenic effect associated with the disease.

## 2 Materials and methods

### 2.1 Reagents

Recombinant human vasoinhibin protein was expressed in *Escherichia coli* (*E. coli*, custom production, Giotto Biotech, Florence, Italy), which comprised the first 147 residues of the mature PRL sequence. A synthetic, cyclic, retro-inverse vasoinhibin heptapeptide (CRIVi45-51), containing the antiangiogenic motif of vasoinhibin (11) (GenScript, Piscataway NJ), was also used.

### 2.2 Human blood samples

Blood samples were collected from pregnant women presenting at the Department of Gynecology and Obstetrics of the General Hospital Nuremberg who were either healthy (n = 9, group “healthy”) or diagnosed with preeclampsia (n = 8) or pregnancy-induced hypertension (PIH) (n = 1) (group “PE” or “Preeclampsia”). As PIH is within the spectrum of hypertensive disorders in pregnancy which are presumed to be vasoinhibin-related in terms of higher circulating levels of vasoinhibin (32), the patient with PIH was not excluded. The samples were taken before delivery of the infants by caesarean section or vaginal delivery. Written informed consent was obtained from the study participants. The samples were aliquoted and stored at −80°C until experimental analysis. Two pools of serum samples were prepared, which included nine healthy pregnancy samples in one pool, and eight preeclampsia patient samples and one PIH patient sample in the second pool.

In agreement with the recommendations of the American College of Obstetricians and Gynecologists Task Force on Hypertension in Pregnancy, the diagnosis of preeclampsia was established when blood pressure was ≥140 mm Hg systolic or ≥90 mm Hg diastolic and was accompanied by proteinuria (≥ 300 mg/24 h), or, in the absence of proteinuria, new onset hypertension was accompanied by thrombocytopenia, renal insufficiency, impaired liver function, pulmonary oedema, and cerebral or visual symptoms (25).

The study was conducted in accordance with the ethical standards of the WMA Declaration of Helsinki and its ethical principles for medical research involving human subjects. Written informed consent has been obtained from the study participants. The study was approved by the ethics committee of the Bavarian Chamber of Physicians on August 29, 2019, and registered at the German Clinical Trials Register, DRKS-ID: DRKS00017719, on December 23, 2019.

### 2.3 Vasoinhibin ELISA

A microplate was coated with 4 µg/ml of vi-mab (31) diluted in carbonate buffer (100 µl/well) overnight at 4°C. The wells were washed with tris-buffered saline-tween (TBST) three times followed by the application of 300 µl of a bovine serum albumin (BSA) blocking solution (Thermo Fisher, cat. No. 37520) for 1 h at room temperature (RT). After washing with TBST, 100 µl of each serum sample (1:32 dilution), or of the vasoinhibin standard in phosphate-buffered saline (PBS), was added to its corresponding well and incubated for 2 h at RT. The plate was washed with TBST followed by the addition of the biotinylated anti-PRL polyclonal antibody (Assay Pro, cat. No. 33110-05121, diluted 1:1000 in blocking buffer), which was incubated for 2 h at RT. The plate was washed, and 100 µl of streptavidin protein conjugated to a polymerized form of horseradish peroxidase enzyme (Poly-HRP, Thermo Fisher, cat. No. 21140, 1:3000 diluted in blocking buffer) was added to each well and incubated for 1 h at RT. The plate was washed, and 100 µl of One Step Ultra TMB ELISA substrate solution (Thermo Fisher, cat. No. 34028) was added to each well and incubated for 15 min. Finally, 100 µl of stop solution (Invitrogen, cat. No. SS04) was added to each well, and the absorbance of each well was read on a plate reader at 450 nm.

### 2.4 Endothelial cell proliferation assay

HUVECs were isolated as described previously (33) and maintained in F12K medium supplemented with 20% fetal bovine serum (FBS), 100 µg/ml heparin (Sigma-Aldrich, St. Louis, MO), and 25 µg/ml of endothelial cell growth supplement (Corning, Inc., Corning, NY) in a humidified 5% CO_2_ atmosphere at 37 °C. HUVECs were seeded in a 96-well plate at 20,000 cells/cm^2^. Twenty four hours later, HUVECs underwent serum starvation with 0.5% FBS-F12K medium supplemented with heparin for 12 h. Next, 25 ng/ml of vascular endothelial growth factor (VEGF, GenScript, Piscataway, NJ) and 20 ng/ml of basic fibroblast growth factor (bFGF, provided by Scios, Inc., Mountain View, CA) were added to the cells either alone or in combination with different concentrations of vasoinhibin produced in *E. Coli* (Giotto Biotech) together with 10 µM of the thymidine analog 5-ethynyl-2’-deoxyuridine (EdU) (Sigma-Aldrich). In some experiments, 4 µg/ml of vi-mab (31) was also added, with the mouse monoclonal beta-actin antibody (Santa Cruz Biotechnology, Dallas, TX) used as control (mouse IgGs), in 20% FBS-F12K medium with heparin.

The optimal sera concentration was determined by testing different dilutions (1:16 or 1:32) of healthy male control serum in 20% FBS-F12K-heparin medium on HUVEC proliferation. The 1:16 dilution was selected and was added to HUVECs in the presence or absence of 25 ng/ml VEGF and 20 ng/ml bFGF either alone or in combination with 1,600 ng/ml vasoinhibin with or without 4 µg/ml vi-mab. After 24 h, the proliferating cells were stained with fluorescent Azide Fluor 545 (Sigma-Aldrich) and the click reaction was assessed (34, 35), and total cells were counterstained with Hoechst (36). Images were obtained with a fluorescence inverted microscope and were quantified using CellProfiler software (37). The proliferation values were interpolated into the linear standard curve of vasoinhibin bioactivity.

### 2.5 CAM assay

The method was performed as previously reported (38). Fertilized eggs from White European Leghorn chicken were obtained from Lohmann Süd GmbH, Dieburg, Germany. They were stored for 24 h at 13°C in an upright position, and then placed in an incubator (HEKA-Brutgeräte, Rietberg, Germany, type “In-Ovo”) at a temperature of 37.7°C, 60% humidity, and two automatic rotations per 24 h. After 72 h, the eggs were cleaned with a 70% ethanol solution and the shell was punctured with forceps in the top position of the egg. The hole was sealed with tape, and the incubation was continued without rotations. On day 5 of the incubation, an opening with the dimensions of 1 x 1 cm was cut into the shell at the position of the hole using a circular saw. The developing CAM can be observed through this opening. The opening was sealed with parafilm, and the incubation was continued. This procedure prevents attachment of the CAM in the upper quadrant of the egg. Incubation was continued at a higher humidity of 70% to prevent drying of the developing CAM. Circular Whatman filter discs containing 6 µl PBS, 1 µg bFGF (PeproTech, Cat. No. 100-18B), 7.5 µg recombinant human vasoinhibin (rhVi), 345 ng CRIVI45-51, 20 µl pooled serum (out of 150 µl pooled serum with 150 µl PBS) from patients with or without preeclampsia, and 20 µl pooled preeclampsia serum with vi-mab (150 µl pooled preeclampsia serum with 130 µl PBS and 20 µl vi-mab at 1.8 mg/ml) with or without a mouse IgG isotype control (Invitrogen, Waltham, MA, cat. No. 02-6502) were placed on the vascularized CAM on day 7 of the incubation. The eggs were incubated until day 10, removed from the incubator, and placed in a stereomicroscope for photographic documentation at 8 X magnification (Science ETD-201 8-50 x, with digital camera MikroCamII 3.1MP, Bresser, Rhede, Germany). The number of blood vessels radially converging and contacting the Whatman filter disc was manually counted (39). Blood vessels with a diameter >100 µm were excluded.

### 2.6 Statistical analyses

Statistical analyses were performed using GraphPad Prism 9 for Windows, Version 9.5.1 (GraphPad Software, Boston, MA). The ELISA curves were fitted by the One Site-Total binding analysis calculated with at least 9 points. The limit of detection (LOD) and quantification were calculated as the mean of the blank signal plus 3 or 10 times, respectively, the standard deviation of the blank. The dose–response curves for the endothelial cell proliferation assay were fitted by least square regression analysis with a variable slope model calculated with at least 9 points. The linear zone of the proliferation assay to quantify the vasoinhibin concentration was determined by a simple linear regression. For the statistical analysis of more than two groups (serum dilutions comparison), a two-way ANOVA followed by the Dunnet test was performed. The overall significance threshold was set at p < 0.001. Radially converging blood vessels in the CAM assay were analyzed by an ordinary one-way ANOVA with the Dunnett’s multiple comparisons test. A p-value <0.05 was considered significant.

## 3 Results

### 3.1 Vasoinhibin ELISA

**Figure 1:**
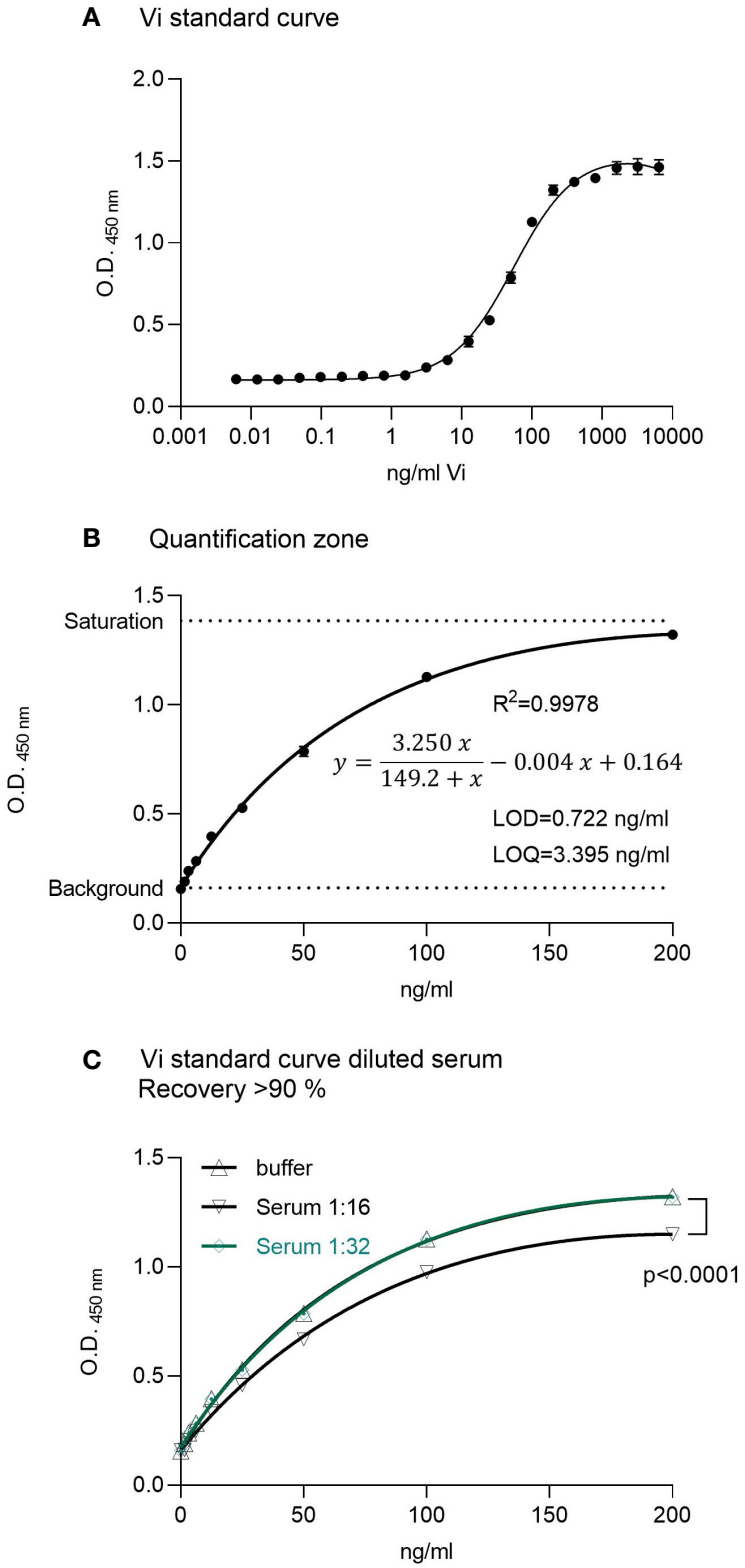
ELISA for vasoinhibin. **(A)** A recombinant vasoinhibin (rhVi) standard produced in *E. Coli*, dissolved in TBST, demonstrated a standard curve with an R² of 0.996. **(B)** Linear range or quantification zone of the ELISA. **(C)** A 1:32 dilution of human serum demonstrated a recovery > 90% of the added standard concentrations. Values are means ± SD, n ≥ 9, p<0.0001 vs. rhVi curve in buffer (two-way ANOVA, Dunnett’s test).

A standard curve using rhVi in TBST buffer was established (**Figure 1A**). The LOD was 0.722 ng/ml, and the limit of quantitation (LOQ) 3.395 ng/ml with a goodness of fit R^2 =^ 0.998 in the quantification zone (**Figure 1B**). A 1:32 dilution of human serum demonstrated a recovery of >90% of the added standard concentrations (**Figure 1C**). The intra-assay and inter-assay coefficients of variation were 2.68% and 3.63% respectively, evaluated at 25 ng/ml with n = 10.

### 3.2 HUVEC proliferation assay for the bioactivity of vasoinhibin

**Figure 2:**
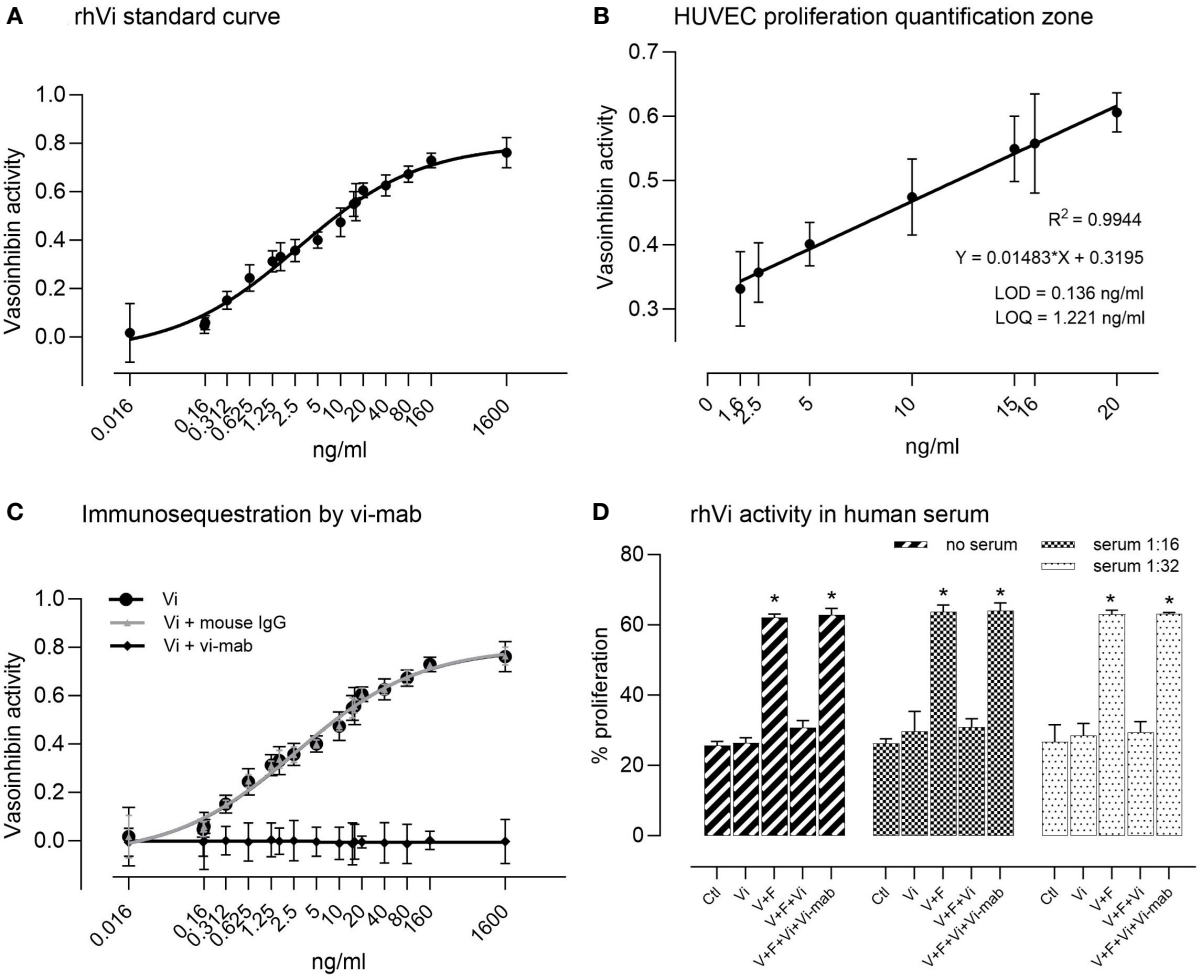
HUVEC proliferation assay for the bioactivity of vasoinhibin. **(A)** The recombinant vasoinhibin (rhVi) standard from *E*. *coli* inhibited HUVEC proliferation in a dose-dependent manner. **(B)** The goodness of fit was R^2 =^ 0.994 in the linear quantification zone determined by a simple linear regression (limit of detection (LOD) = 0.136 ng/ml, LOD = 1.221 ng/ml). **(C)** Co-incubation with the vasoinhibin monoclonal antibody (vi-mab) neutralized the inhibiting effect of vasoinhibin, while the addition of mouse immunoglobulin (IgG) had no effect. Values are means ± SD, n = 9. **(D)** Serial serum dilutions and their evaluation in the endothelial cell proliferation assay demonstrated that added rhVi is active in human serum, and that its activity is adequately evaluated at 1:16 and 1:32 dilutions. V+F: proangiogenic factors VEGF+bFGF. Values are means ± SD, n ≥ 5, *p<0.001 vs. control treatment of each group (two-way ANOVA, Tukey’s test).

The rhVi standard inhibited HUVEC proliferation in a dose-dependent manner (**Figure 2A**), with a goodness of fit R^2 =^ 0.994 in the linear quantification zone (LOD = 0.136 ng/ml, LOQ = 1.221 ng/ml, **Figure 2B**). The addition of vi-mab neutralized the vasoinhibin inhibition effect, while the addition of mouse IgG had no effect (**Figure 2C**). Serial serum dilutions and their evaluation in the endothelial cell proliferation assay demonstrated that the rhVi is active in human serum, and that its activity is adequately evaluated at 1:16 and 1:32 dilutions, where the serum components do not interfere with the VEGF+bFGF stimulation or vasoinhibin activity (**Figure 2D**). The intra-assay and inter-assay coefficients of variation were 2.65% and 7.20% respectively, evaluated at 20 ng/ml with n = 9.

### 3.3 Serum levels of immunoreactive and bioactive vasoinhibin are higher in preeclampsia

**Figure 3:**
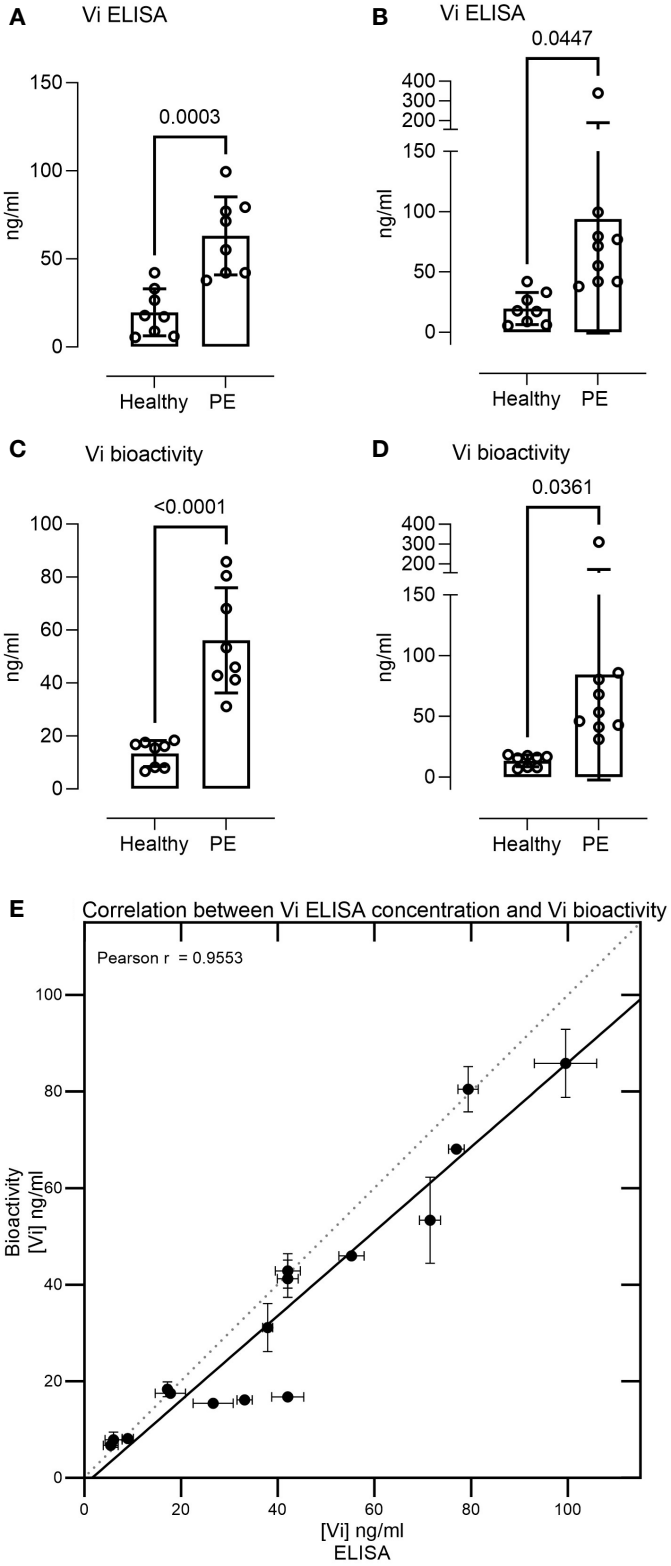
Serum levels of immunoreactive and bioactive vasoinhibin are higher in preeclampsia. The vasoinhibin (Vi) serum concentrations of 17 patients, as determined by ELISA **(A, B)** and the HUVEC (human umbilical vein endothelial cell) proliferation assay for vasoinhibin bioactivity **(C, D)**, with and without an outlier, respectively, are shown. The concentrations ranged between 5 and 340 ng/ml, and significant differences were observed between the healthy and the preeclamptic (PE) samples (Student’s t-test, values are means ± SD, n ≥ 8). **(E)** There was a correlation between the vasoinhibin concentrations measured by the ELISA and the vasoinhibin concentrations determined in the HUVEC proliferation assay (Pearson r = 0.95, p = < 0.0001, excluding the outlier).

Measurement of 17 serum samples from healthy pregnant women and pregnant women with preeclampsia demonstrated vasoinhibin serum concentrations in the range of 5–340 ng/ml. The levels were significantly higher in preeclampsia sera than in healthy pregnancy sera when measured by ELISA (mean 63.09 ± 22.15 SD vs. 19.67 ± 13.34 ng/ml, p = 0.0003, or 93.86 ± 94.61 vs. 19.67 ± 13.34, p = 0.0447 when including one outlier, **Figures 3A, B**). Likewise, preeclampsia sera demonstrated higher levels of vasoinhibin in the HUVEC proliferation assay, compared to healthy pregnancy sera (56.12 ± 19.83 ng/ml vs. 13.38 ± 4.88 ng/ml, p < 0.0001, or 84.38 ± 86.78 ng/ml vs. 13.38 ± 4.88 ng/ml, p = 0.0361 if the outlier is included, **Figures 3C, D**). The correlation between the concentration of vasoinhibin measured by ELISA and by the HUVEC proliferation assay was significant (Pearson r = 0.9553, p < 0.0001, excluding the outlier, **Figure 3E**).

### 3.4 Vasoinhibin in preeclamptic serum inhibits angiogenesis in the CAM assay

**Figure 4:**
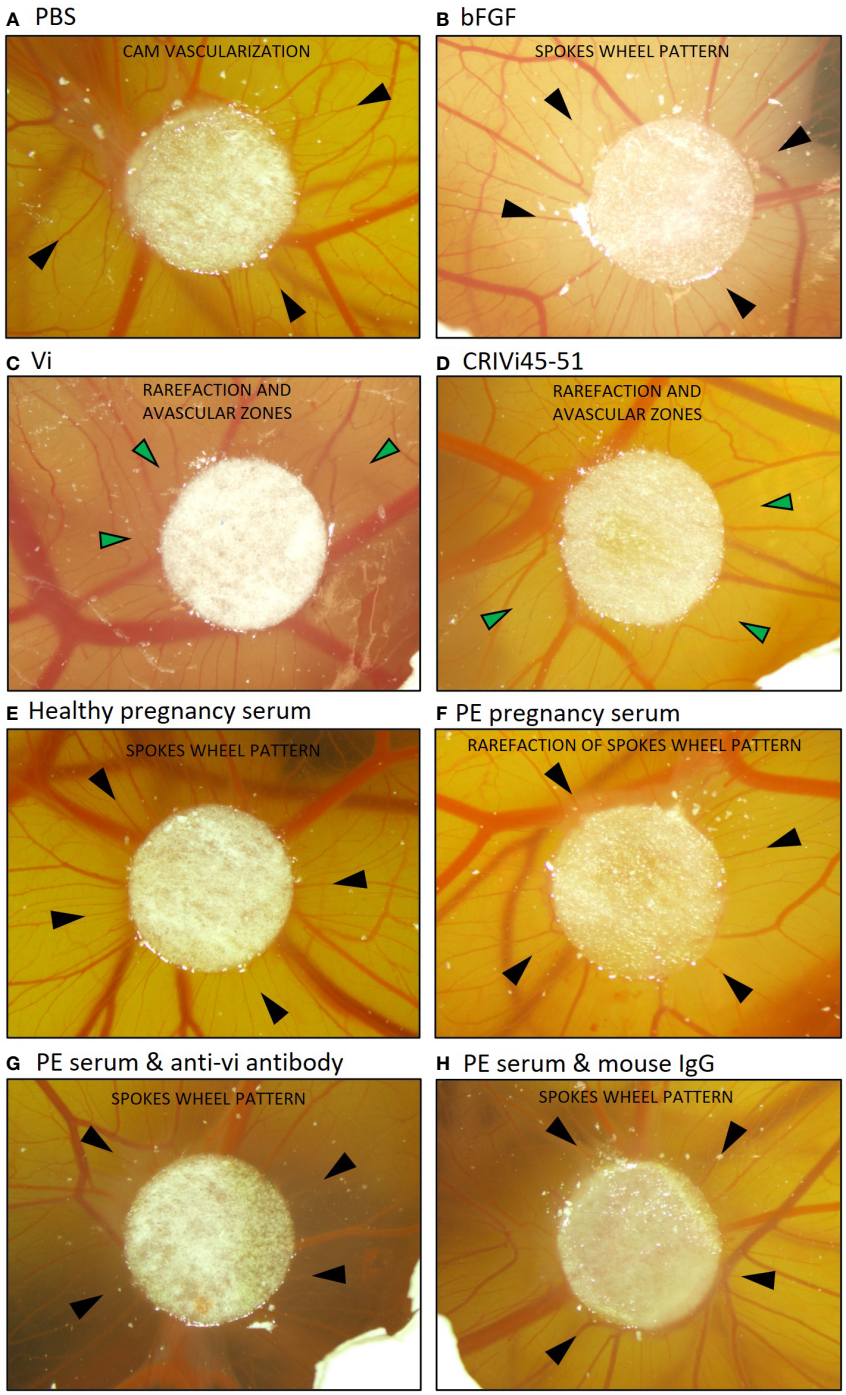
Vasoinhibin in preeclamptic serum inhibits angiogenesis in the CAM assay. Representative images of a vascularized chick chorioallantoic membrane (CAM) at day 10 after treatment with the indicated substance applied on a circular Whatman filter are shown. Black arrows point toward small blood vessels (microvessels) and green arrows toward avascular zones. **(A)** The PBS-treated CAM demonstrated a blood vessel pattern with no apparent relation to the Whatman filter. **(B)** The bFGF-treated CAM demonstrated a spokes wheel-like pattern, in which microvessels radially converge toward the Whatman filter. **(C)** A rarefaction of radial microvessels with larger avascular zones was observed when the CAM was treated with recombinant vasoinhibin (rhVi) or **(D)** CRIVi45-51, a vasoinhibin circular analog. **(E)** Serum from healthy pregnant women induced a spokes wheel-like pattern with an increase in radial microvessels. **(F)** Radial microvessels in the CAM treated with serum from patients with preeclampsia are at PBS level, and **(G)** increase after treatment with vi-mab. **(H)** Serum from patients with preeclampsia enriched with mouse IgG is comparable to preeclampsia serum alone. PBS, phosphate buffered saline; bFGF, basic fibroblast growth factor; Vi, vasoinhibin; CRIVi45-51, cyclic retroinverse vasoinhibin; PE, preeclampsia; IgG, mouse immunoglobulin G.

**Figure 5:**
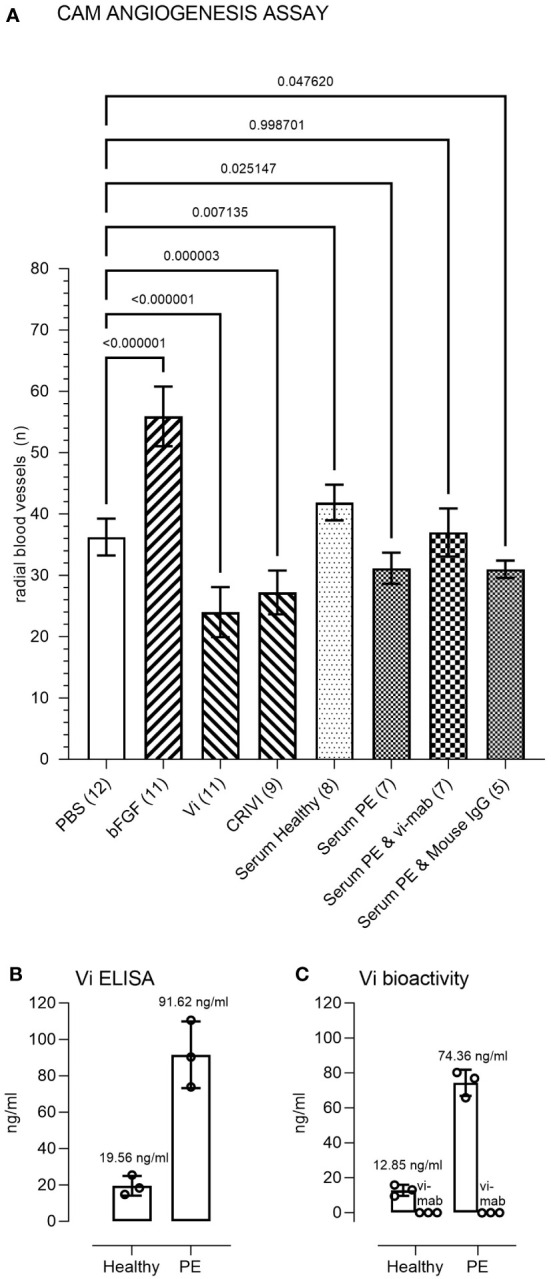
Immunometric and functional evaluation of pooled sera. **(A)** Quantitation and statistical analysis of the angiogenesis response in the chick chorioallantoic membrane (CAM) assay. Comparisons of the number of radial microvessels were made between treatment with PBS and the indicated test substances. The p-values between the groups are indicated over each bracket. The number of CAMs in each group is indicated in brackets after the group designation. PBS, phosphate buffered saline; bFGF, basic fibroblast growth factor; Vi, vasoinhibin; CRIVi45-51, cyclic retroinverse vasoinhibin; PE, preeclampsia pooled serum; IgG, mouse immunoglobulin (G). **(B)** Measurement of Vi levels by ELISA and by the HUVEC proliferation assay **(C)** of the healthy and preeclampsia pooled sera. The vasoinhibin activity in both serum pools was < limit of detection (LOD) when the serum was co-incubated with the vasoinhibin monoclonal antibody (vi-mab). Values are means ± SD.

Pooled preeclampsia sera with and without the ELISA antibody, vi-mab, and healthy pregnancy sera, as well as PBS, bFGF, rhVi, and CRIVi45-51, were evaluated in the CAM angiogenesis assay. **Figure 4** shows micrographs of representative CAMs and **Figure 5A** shows the quantitation of the angiogenic response determined by the radial increase in the number of capillary blood vessels directed toward the implant (39). An increase in radial blood vessel growth was observed following treatment with bFGF, compared to the PBS control (55 ± 4 vs. 36 ± 3 microvessels directed toward the implant, p < 0.0001). Recombinant vasoinhibin (24 ± 4) and synthetic CRIVi45-51 (27 ± 3) inhibited (p < 0.0001) blood vessel growth, compared to the PBS control. A higher number of radial blood vessels was also observed after treatment with the pooled serum from healthy pregnant women (41 ± 2 vs. 36 ± 3, p < 0.05 vs. PBS). In contrast, pooled serum from pregnant patients with preeclampsia significantly reduced microvessel growth compared to the PBS control (31 ± 3 vs. 36 ± 3, p < 0.05). Pretreatment of the preeclampsia serum pool with vi-mab abolished the inhibitory effect, with the number of blood vessels being comparable to those in the PBS control (37 ± 3, p > 0.05). The addition of purified IgGs from mouse serum did not significantly alter the blood vessel count after the preeclampsia serum pool. The vasoinhibin concentration measured by ELISA was 19.56 ng/ml in the pooled serum samples from healthy pregnant patients and 91.62 ng/ml in the pooled serum from the patients with preeclampsia (**Figure 5B**). The HUVEC proliferation assay measured a vasoinhibin level of 13.08 ng/ml in the pooled serum from healthy patients and 74.36 ng/ml in the pooled serum from patients with preeclampsia (**Figure 5C**). The vasoinhibin activity in both serum pools was < LOD when the serum was co-incubated with the ELISA vi-mab (**Figure 5C**).

## 4 Discussion

The present study describes a novel, quantitative, and sensitive ELISA for measuring vasoinhibin levels in the human circulation. We observed higher levels of vasoinhibin in sera from women with preeclampsia than from a healthy pregnancy control group. The specificity of the ELISA is supported by its strong correlation with the levels of bioactive vasoinhibin quantified using the HUVEC proliferation assay. Furthermore, the CAM angiogenesis assay supports the contribution of vasoinhibin to the antiangiogenic properties of the serum, which is characteristic of preeclampsia (40). The essential component of the ELISA is the vi-mab having no cross-reactivity with PRL (**Supplementary Figure S1**) (31). This non-commercial antibody was employed as a capture antibody in a sandwich ELISA format, and a commercial, polyclonal, biotinylated anti-PRL antibody was used as the detection antibody. The vi-mab has not been tested in other applications, such as immunohistochemistry. The polyclonal anti-PRL antibody likely constitutes an interchangeable element of the assay, as it binds vasoinhibin due to shared epitopes with full-length PRL; multiple such antibodies are commercially available.

In this respect, the present study reports, for the first time, that the vasoinhibin concentration ranges between 5 and 340 ng/ml in healthy pregnant women and pregnant women with preeclampsia. This is consistent with the principles of vasoinhibin regulation demonstrated previously (15), where vasoinhibin is generated from PRL stored in secretory granules in the pituitary gland (14) and at the target tissue level with the local cleaving enzyme activity being the key regulatory element. The local generation of vasoinhibin is impacted by the levels of PRL produced and secreted by the pituitary gland into the circulation. Such impact is the basis of clinical trials for the treatment of peripartum cardiomyopathy and diabetic retinopathy, where pharmacological interventions at the pituitary level with a dopamine D2 agonist (bromocriptine) or antagonist (levosulpiride) leading to hypoprolactinemia or hyperprolactinemia, respectively, are evaluated for the inhibition and the stimulation of vasoinhibin generation (41–43).

The specificity of the ELISA is supported by the ability of the vi-mab, but not control antibodies, to block vasoinhibin-induced inhibition of HUVEC proliferation. Furthermore, the vi-mab blocked the inhibition of HUVEC proliferation by the endogenous hormone in serum. In addition, the vasoinhibin levels measured by the ELISA and the HUVEC bioassay were highly correlated (Pearson r = 0.9553, p < 0.0001), and similar values were quantified in healthy pregnancy samples (19.56 ± 5.40 ng/ml vs. 12.85 ± 3.18 ng/ml, p = 0.1347) and in preeclampsia samples (91.62 ± 18.37 ng/ml vs. 74.36 ± 7.53 ng/ml, p = 0.2064), indicating that circulating vasoinhibin is upregulated in preeclampsia.

Antiangiogenic factors released by the placenta, such as soluble VEGF receptor 1 (sFlt1) which antagonizes the proangiogenic proteins PlGF and VEGF in the maternal circulation, are central to the pathophysiology of preeclampsia (44–46). Previous studies have claimed vasoinhibin to be one of these factors (7, 8, 23, 27). Immunoprecipitation and Western blotting analyses showed upregulation of vasoinhibin levels in the amniotic fluid, serum, and urine of preeclamptic women due to cathepsin D-mediated cleavage of PRL by the placenta (8, 27). However, the limited quantitative value and insufficient detection sensitivity of such techniques limits their reliability and further exploration of the findings. The current vasoinhibin ELISA supports these observations and provides the unique opportunity to investigate the function and upregulation of vasoinhibin in preeclampsia.

The CAM assay demonstrated the proangiogenic effects and antiangiogenic effects of serum from healthy pregnancy and preeclampsia patients, respectively, and further implicated vasoinhibin as an antiangiogenic factor contributing to this difference. To our knowledge, this is the first study evaluating the effect of serum from patients with preeclampsia in the CAM assay. The observed differences in angiogenic capacity are consistent with previous studies showing that lower levels of placental growth factor (PlGF) and VEGF and higher levels of sFlt1 determine the antiangiogenic properties of serum in preeclampsia (40, 47). Virtanen et al. used co-cultures of HUVECs and fibroblasts or HUVECs and human adipose stromal cells (hASCs) to demonstrate the inhibitory effects of systemic serum and umbilical cord blood sera from patients with preeclampsia on angiogenesis and vasculogenesis (40, 48).

The absence of an antiangiogenic effect with serum from healthy pregnancy patients is consistent with the growing pregnancy state and suggests that the action of antiangiogenic effectors, including vasoinhibin, is counteracted by an overall proangiogenic environment (40). In contrast, in preeclampsia, there is a shift toward a net antiangiogenic environment, which likely involves vasoinhibin. This is consistent with previous work showing that vasoinhibin in the amniotic fluid from preeclamptic pregnancy inhibits VEGF-induced proliferation of cultured endothelial cells (8).

A limitation of this study is that it is not a case–control study; although significant p values substantiate conclusions, the number of patients is small and the study does not comply with the standards required for reporting case–control studies (49). In addition, there was no evaluation of the suitability of the vasoinhibin level as a biomarker for risk stratification, screening, or diagnostic purposes. However, since the technical development of the ELISA for human vasoinhibin is the core element of the study, and the evaluation of serum samples of healthy pregnant women and women with preeclampsia was performed to demonstrate a relevant clinical applicability, these limitations appear acceptable within the scope of this study. Nevertheless, the vasoinhibin level distribution across both groups of patients and its function in regulating vessel growth in the CAM and HUVEC endothelial cell proliferation assay form a convincing case supporting further technical and clinical verification.

In summary, the present study reports the development of an immunometric assay for the quantitative determination of endogenous vasoinhibin in human serum and supports the upregulation of vasoinhibin concentrations in serum from preeclamptic women. Furthermore, it provides further evidence that vasoinhibin in the serum exerts antiangiogenic effects in women with preeclampsia, but not in women with a healthy pregnancy, perhaps due to a counteracting proangiogenic environment. The development and implementation of a quantitative immunometric assay for vasoinhibin overcomes a long-standing barrier and enables the future perspective of the thorough clinical verification of vasoinhibin as a relevant biomarker.

## Author’s note

The content of this manuscript has been presented in part at the Society for Endocrinology BES 2023 Conference in Glasgow, UK, November 13-15, 2023 and published in *Endocrine Abstracts* (2023) 94 P111, DOI: 10.1530/endoabs.94.P111.

## Data availability statement

The raw data supporting the conclusions of this article will be made available by the authors, without undue reservation.

## Ethics statement

The studies involving humans were approved by the ethics committee of the Bavarian Chamber of Physicians on August 29, 2019, and registered at the German Clinical Trials Register, DRKS-ID: DRKS00017719 on December 23, 2019. The studies were conducted in accordance with the local legislation and institutional requirements. The participants provided their written informed consent to participate in this study. Ethical approval was not required for the studies on animals in accordance with the local legislation and institutional requirements because only commercially available established cell lines were used.

## Author contributions

MZ: Conceptualization, Data curation, Formal analysis, Funding acquisition, Investigation, Methodology, Project administration, Software, Validation, Visualization, Writing – review & editing. DH: Conceptualization, Data curation, Formal analysis, Investigation, Methodology, Software, Validation, Visualization, Writing – review & editing. ND: Data curation, Formal analysis, Investigation, Methodology, Validation, Conceptualization, Writing – review & editing. JE: Data curation, Formal analysis, Investigation, Methodology, Validation, Conceptualization, Writing – review & editing. PR: Conceptualization, Project administration, Supervision, Writing – review & editing. CB: Conceptualization, Project administration, Supervision, Validation, Writing – review & editing. CW: Conceptualization, Project administration, Supervision, Validation, Writing – review & editing. JPR: Conceptualization, Data curation, Formal analysis, Investigation, Methodology, Supervision, Validation, Writing – review & editing. TB: Conceptualization, Data curation, Formal analysis, Funding acquisition, Investigation, Methodology, Project administration, Resources, Supervision, Validation, Visualization, Writing – review & editing. CC: Conceptualization, Data curation, Formal analysis, Investigation, Methodology, Project administration, Supervision, Validation, Writing – review & editing, Resources, Funding acquisition, Visualization. JT: Conceptualization, Data curation, Formal analysis, Funding acquisition, Investigation, Methodology, Project administration, Resources, Supervision, Validation, Visualization, Writing – original draft.
