# Supplementary material for: Immunometric and functional measurement of endogenous vasoinhibin in human sera
Source: Front Endocrinol (Lausanne). 2024 Apr 29;15:1345996. doi: 10.3389/fendo.2024.1345996 (PMC11089174; doi:10.3389/fendo.2024.1345996)
Supplement: Supplementary file 1 [file DataSheet_1.pdf]

Supplementary Material

Immunometric and Functional Measurement of Endogenous Vasoinhibin in Human Sera

Magdalena Zamora & David Harris et al. 2024

Corresponding Author: Jakob Triebel; Jakob.Triebel@gmx.de **Supplementary**

Figure

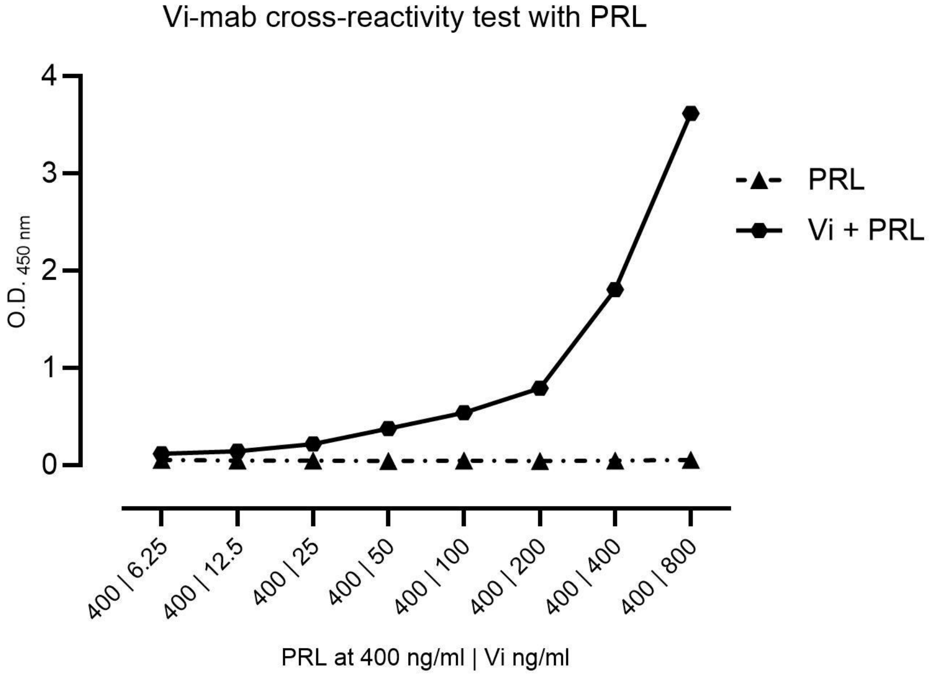

Supplementary Figure 1. The ELISA with the vasoinhibin monoclonal antibody (Vi-mab) was evaluated for cross-reactivity with prolactin (PRL) by measuring PRL alone and in the presence of various concentrations of recombinant vasoinhibin. The reactivity of PRL alone was at blank level, and it did not interfere with the detection of vasoinhibin (Vi) when measured in combination.
